# How Do Nigeria's Health-Care Personnel Treat Patients with HIV/AIDS?

**DOI:** 10.1371/journal.pmed.0020257

**Published:** 2005-08-19

People living with HIV/AIDS (PLWA) face many forms of stigma and discrimination. This is the case in whichever country they may live, as has been shown in a number of previous research studies. In addition to experiencing unfair treatment in their families, communities, and places of work, PLWA may encounter discrimination from health-care professionals. This can interfere with effective prevention and treatment. Discriminatory practices in the health-care sector may also appear to legitimize other forms of discrimination against PLWA.

Vincent Iacopino and colleagues from the organization Physicians for Human Rights, in collaboration with researchers from Policy Project–Nigeria and the Center for the Right to Health (also in Nigeria) investigated the problem in Nigeria. With a population of roughly 130 million, Nigeria is home to one in 11 of the 40 million PLWA worldwide. Around 6% of adult Nigerians are thought to be HIV-positive, and there will be an estimated 310,000 AIDS deaths this year. The indications are that infection rates will increase. Until now, little has been known about the nature and extent of discrimination against patients with HIV/AIDS in Nigeria.

Trained interviewers conducted a cross-sectional questionnaire survey of 1,021 Nigerian health-care professionals in 111 health-care facilities in four of Nigeria's 36 states. Those sampled were 324 physicians, 541 nurses, and 133 midwives, and 23 health-care workers of unknown profession. Fifty-four percent of them worked in public tertiary care facilities. Many of the survey's results are worrying. Nine percent of professionals reported refusing to care for a patient with HIV/AIDS, and 9% said they had refused a patient with HIV/AIDS admission to a hospital. Fifty-nine percent agreed that PLWA should be on a separate ward, and 40% believed a person's HIV status could be determined by their appearance. Ninety-one percent agreed that staff should be informed when a patient was HIV-positive in order to protect themselves. Forty percent believed health-care professionals with HIV/AIDS should not be allowed to work in any area of health-care requiring patient contact. Twenty percent agreed that many with HIV/AIDS had behaved immorally and deserved their infection. Eight percent felt that treating someone with HIV/AIDS was a waste of resources.

Providers who reported working in facilities that did not always practice universal precautions against HIV transmission were more likely to favor restrictive policies towards PLWA. In general, basic materials needed for treatment and prevention of HIV infection were not sufficiently available. Providers who reported less adequate training in HIV/AIDS treatment and in ethics were more likely to report negative attitudes towards patients with HIV/AIDS. There was no consistent pattern of differences in negative attitudes and practices across the different professions surveyed.[Fig pmed-0020257-g001]

**Figure pmed-0020257-g001:**
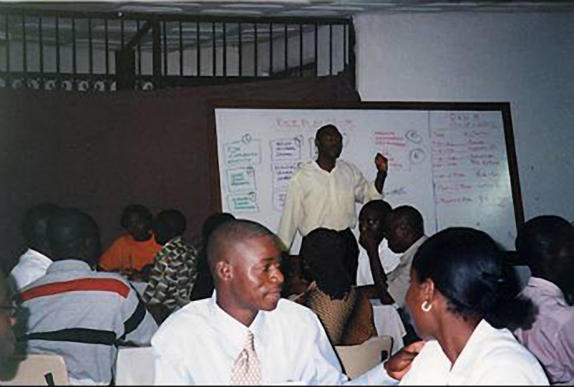
Training of interviewers for the study included 5 days of classroom teaching and role-playing

The researchers concluded that, while most health-care professionals surveyed reported being in compliance with their ethical obligations, discriminatory behavior and attitudes towards patients with HIV/AIDS existed among a significant proportion. Inadequate education about HIV/AIDS and a lack of protective and treatment materials appear to favor these practices and attitudes. The findings of the study, in just four states, cannot be generalized to Nigeria as a whole and, although sampled systematically, it is possible that sampled facilities and health-care professionals may differ significantly from those that were not sampled in the study states. Concerns over a perceived lack of privacy in the interviews or about job status may have resulted in an underreporting of discriminatory behavior and/or an overreporting of “correct” practices or attitudes. The authors note that the health-care system in Nigeria is underfunded and suffers from fundamental problems, including material scarcity and inadequacies in infrastructure, both of which may contribute to discriminatory behavior. They call for targeted education of health-care professionals and provision of adequate resources to health-care facilities, and for the introduction and enforcement of anti-discrimination policies.

